# Supplementary material for: Determination of Rhodamine 6G with direct immersion single-drop microextraction combined with an optical probe
Source: PLoS One. 2024 Aug 19;19(8):e0309121. doi: 10.1371/journal.pone.0309121 (PMC11332950; doi:10.1371/journal.pone.0309121)
Supplement: S1 File — (DOCX) [file pone.0309121.s001.docx]

**S1 Table. AGREE assessment of the procedures used for Rh6G determination combining microextraction and spectrophotometry: combination of an optical probe and single-drop direct immersion microextraction (this work), magnetic stirring assisted dispersive liquid–liquid microextraction [7], derivative ratio spectrophotometry after dispersive liquid–liquid microextraction [10], hollow ﬁber liquid-phase microextraction [19].**

| AGREE parameters | Developed method | | Method [7] | | Method [10] | | Method [19] | |
| --- | --- | --- | --- | --- | --- | --- | --- | --- |
|  | Comments | Score | Comments | Score | Comments | Score | Comments | Score |
| 1. Direct Analytical Techniques Should Be Applied to Avoid Sample Treatment | At-line analysis | 0.6 | Off-line analysis | 0.48 | External sample pre- and treatment and batch analysis (reduced number of steps) | 0.30 | External sample pre- and treatment and batch analysis (large number of steps) | 0.0 |
| 2. Minimal Sample Size and Minimal Number of Samples Are Goals | 7.5 mL | 0.37 | 50 mL | 0.094 | 8 mL | 0.35 | 50 mL | 0.094 |
| 3. In Situ Measurements Should Be Performed | At-line analysis | 0.33 | Off-line analysis | 0.0 | Off-line analysis | 0.0 | Off-line analysis | 0.0 |
| 4. Integration of Analytical Processes and Operations Saves Energy and Reduces the Use of Reagents | Two steps | 1.0 | Five steps | 0.6 | Four steps | 0.8 | Five steps | 0.6 |
| 5. Automated and Miniaturized Methods Should Be Selected | Semi-automatic, miniaturized | 0.75 | Manual, miniaturized | 0.5 | Manual, miniaturized | 0.5 | Manual, miniaturized | 0.5 |
| 6. Derivatization Should Be Avoided | Picric acid is used to form IA | 0.8 | – | 1.0 | – | 1.0 | – | 1.0 |
| 7. Generation of a Large Volume of Analytical Waste Should Be Avoided and Proper Management of Analytical Waste Should Be Provided | 7.5 mL | 0.43 | 50 mL | 0.17 | 8 mL | 0.41 | 50 mL | 0.17 |
| 8. Multianalyte or Multiparameter Methods Are Preferred versus Methods Using One Analyte at a Time | 2 h^–1^ | 0.12 | 6 h^–1^ | 0.38 | 12 h^–1^ | 0.55 | 4 h^–1^ | 0.28 |
| 9. The Use of Energy Should Be Minimized | UV-Vis Spectrometry | 1.0 | HPLC | 0.5 | UV-Vis Spectrometry | 1.0 | HPLC | 0.5 |
| 10. Reagents Obtained from Renewable Source Should Be Preferred | None of the reagents originates from bio-based sources | 0.0 | None of the reagents originates from bio-based sources | 0.0 | None of the reagents originates from bio-based sources | 0.0 | None of the reagents originates from bio-based sources | 0.0 |
| 11. Toxic Reagents Should Be Eliminated or Replaced | 55 µL of amyl acetate | 1.0 | 500 µL of acetone, 1.05 mL of n-octanol | 0.53 | 2.5 mL of ethanol, 250 µL of chloroform | 0.43 | 25 µL of 1-octanol | 1.0 |
| 12. The Safety of the Operator Should Be Increased | Toxic to aquatic life | 0.8 | Toxic to aquatic life, highly flammable | 0.6 | Toxic to aquatic life, persistent | 0.6 | Toxic to aquatic life | 0.8 |
| **The overall score** |  | **0.58** |  | **0.4** |  | **0.49** |  | **0.4** |
