# Supplementary material for: Determination of Rhodamine 6G with direct immersion single-drop microextraction combined with an optical probe
Source: PLoS One. 2024 Aug 19;19(8):e0309121. doi: 10.1371/journal.pone.0309121 (PMC11332950; doi:10.1371/journal.pone.0309121)
Supplement: Attachment — Submitted filename: Response to Reviewers.docx [file pone.0309121.s003.docx]

Responses to Reviewer's questions

Minor issues:
1. Lines 160-161: This sentence has no context? Something left out?
A previous review indicated that the manuscript needed to be corrected to meet PLOS ONE`s style requirements. The following was suggested. 2. In your Methods section, please provide additional information regarding the permits you obtained for the work. Please ensure you have included the full name of the authority that approved the field site access and, if no permits were required, a brief statement explaining why. The text of the manuscript has therefore been finalized to reflect this suggestion.

2. Line 203: Change to “salting out”

The text has been corrected in line with this comment.

Major issues:
1. In Table 1, the units are mM, in Table 2, nM, in Table 3 µg L-1. For readers who do not wish to look up the molar or atomic mass of something in order to convert between units, this is troublesome and irritating. It also makes it difficult to recognize the significance of 10 mM of carbonate in river water, compared to the linear range of 5-240 µg L-1 for your method without conversion to identical units.
Concentration units in the article (Table 3, abstract and Introduction) were converted to one unit system derived from mol L^–1^ (nM or mM).

2. In Table 3 it would be useful for comparison of methods to know, without having to go to the original publications, the volume of sample and final analytical instrumentation technique, to again accurately compare the significant differences in methodology and greenness.

The volume of sample has been added to Table 3. The caption to Table 3 indicates the final analytical instrumental methodology. In all compared methods, the analytical signal was measured by spectrophotometry.

3. For Figure 6, there should be a listing of the 4 methods in the footnote to identify each AGREE assessment.

4 methods have been listed in the caption to Fig. 6.

4. For Figure 6, it would be helpful to the reader and context of the figure, to provide the calculations for each AGREE assessment. This would be best handled in the electronic supplemental, referenced in the Table and manuscript of course.

S1 Table has been added and posted as Supporting Information. It contains the data needed to assess the AGREE score for the developed and three compared methods. The AGREE scores have been refined.
